# Design for a multicenter, randomized, sham-controlled study to evaluate safety and efficacy after treatment with the Nuvaira® lung denervation system in subjects with chronic obstructive pulmonary disease (AIRFLOW-3)

**DOI:** 10.1186/s12890-020-1058-5

**Published:** 2020-02-13

**Authors:** Dirk-Jan Slebos, Bruno Degano, Arschang Valipour, Pallav L. Shah, Gaetan Deslée, Frank C. Sciurba, on behalf of the AIRFLOW-3 Trial Study Group

**Affiliations:** 1 Department of Pulmonary Diseases, University Medical Center Groningen, University of Groningen, Hanzeplein 1, 9713RB, Groningen, The Netherlands; 2 Department of Respiratory Medicine, CHU de Grenoble Alpes, Grenoble, France; 3 Department of Respiratory and Critical Care Medicine, Karl-Landsteiner-Institute for Lung Research and Pulmonary Oncology, Krankenhaus Nord-Klinik Floridsdorf, Vienna, Austria; 4 Royal Brompton & Harefield NHS Trust, Chelsea & Westminster Hospital and Imperial College, London, UK; 5 CHU de REIMS, Hôpital Maison Blanche Service des Maladies Respiratoires INSERM 1250, 45 rue Cognacq Jay, 51092 Reims Cedex, France; 6 University of Pittsburgh School of Medical Center, 200 Lothrop Street, Pittsburgh, PA 15213 USA

**Keywords:** COPD, Nerves, Targeted lung denervation, Acetylcholine, Anticholinergic, Bronchoscopy

## Abstract

**Background:**

Targeted lung denervation (TLD) is a bronchoscopically delivered ablation therapy that selectively interrupts pulmonary parasympathetic nerve signaling. The procedure has the potential to alter airway smooth muscle tone and reactivity, decrease mucous secretion, and reduce airway inflammation and reflex airway hyperresponsiveness. Secondary outcome analysis of a previous randomized, sham-controlled trial showed a reduction in moderate-to-severe exacerbations in patients with COPD after TLD treatment. A pivotal trial, AIRFLOW-3 has been designed to evaluate the safety and efficacy of TLD combined with optimal medical therapy to reduce moderate or severe exacerbations throughout 1 year, compared with optimal medical therapy alone.

**Methods:**

The study design is a multicenter, randomized, full sham bronchoscopy controlled, double-blind trial that will enroll 400 patients (1:1 randomization). Key inclusion criteria are FEV_1_/FVC < 0.7, FEV_1_ 30 to 60% of predicted, post-bronchodilator, ≥ 2 moderate or 1 severe COPD exacerbations in the prior year, and COPD assessment test (CAT) ≥ 10. Primary objective will be the comparison of moderate or severe COPD exacerbations through 12 months of TLD therapy with optimal medical therapy versus optimal medical therapy alone. The sham group will be allowed to cross over at 1 year. Patients will be followed for up to 5 years.

**Discussion:**

The multicenter, randomized, full sham bronchoscopy controlled, double-blind AIRFLOW-3 trial will evaluate the efficacy of TLD to reduce moderate or severe COPD exacerbations beyond optimal medical therapy alone. The target population are patients with COPD, who suffer persistent symptoms and exacerbations despite optimal treatment, defining an unmet medical need requiring novel therapeutic solutions. This trial is registered at clinicaltrials.gov: NCT03639051.

## Background

COPD is characterized by persistent respiratory symptoms and airflow limitation due to airway and/or alveolar abnormalities [1]. COPD is a major cause of morbidity and mortality worldwide, and in the United States, costs associated with hospitalizations for exacerbations represent the largest proportion of total cost across all disease stages [2]. Reducing the risk for future exacerbations is a crucial guideline-directed goal of COPD management [1].

Inhaled pharmacologic treatments for COPD include drugs stimulating adrenergic receptors in airway smooth muscle (long-acting ß-agonists; LABA) or preventing acetylcholine binding to muscarinic receptors in the airways (long-acting muscarinic antagonists; LAMA) to induce bronchodilation, relax airway smooth muscle, and reduce airway inflammation [3]. LABA and LAMA also reduce exacerbation risk, and adding inhaled corticosteroids in dual or triple therapy may also enhance this effect in some patients [1, 4]. However, despite the benefits of inhaled pharmacologic treatments for COPD, a significant number of patients have persistent symptom and exacerbation burden (classified as GOLD Group “D”). Development of a therapeutic procedure that could reduce the risk for future exacerbation is an important research objective [5].

Baseline autonomic input of the vagus nerve, which modulates airway smooth muscle tone, mucus hypersecretion and hyperresponsiveness [6–8], is elevated in COPD [6]. Targeted lung denervation (TLD) aims to disrupt pulmonary nerve input to and from the lung to reduce clinical consequences of neural hyperactivity via improved bronchodilation, reduced mucous secretion of airway submucosal glands, and reduced airway hyperresponsiveness through disruption of pulmonary nerve reflexes [6–8]. Other potential impacts of TLD include disruption of other mediators of mucous secretion and inflammation such as neuropeptides [9]. Previous studies of TLD therapy have demonstrated proof of concept, evaluated dosing, established a safety profile, and identified potential efficacy outcomes [10–12]. Secondary analysis of AIRFLOW-2, a phase IIB safety multicenter study using a 1:1 randomized, sham-controlled, double-blinded design showed a statistically significant decrease in hospitalizations for COPD exacerbation with a trend toward significance for moderate-to-severe exacerbations [11]. Given these promising results, a prospective study of TLD therapy in a larger group of patients is warranted.

This paper describes the study design for A Multicenter, Randomized, Sham-controlled Study to Evaluate Safety and Efficacy After Treatment with the Nuvaira® Lung Denervation System in Subjects with Chronic Obstructive Pulmonary Disease (AIRFLOW-3). The primary objective of this study is to evaluate the efficacy of TLD to reduce moderate or severe (hospitalized) exacerbations of COPD beyond optimal medical therapy alone.

## Methods/design

### Overview

AIRFLOW-3 is a prospective, multicenter, randomized, sham-controlled, double-blind, safety and efficacy study designed to prospectively evaluate TLD’s impact on moderate-to-severe exacerbations in GOLD Group D patients. The AIRFLOW-3 subject profile is diagnosed COPD with FEV_1_ 30–60% predicted, a documented history of at least 2 moderate or 1 severe exacerbation in the 12 months prior to consent, and persistent symptoms (CAT > 10) while on optimal medical treatment [1]. Up to 40 academic investigational centers are planned (approximately 25 US sites (> 60% subject participation)) and 15 sites in Europe (France, UK, Netherlands, Germany, Austria) and Canada (< 40% subject participation). Participants will be randomized (1:1) to TLD therapy plus optimal medical care (active treatment) or sham bronchoscopy procedure plus optimal medical care (sham control) utilizing a clinical electronic data capture (EDC) software. Randomization will be stratified based on site, participation in a pulmonary rehabilitation maintenance program, and baseline use of an inhaled corticosteroid at the time of enrollment. Stratification, which normalizes the impact of ICS and PR on patient outcomes, has no impact on the statistical power of the trial. The study is registered on clinicaltrials.gov (NCT03639051) and the protocol will have site ethics committee (EC) or Institutional Review Board (IRB) approval prior to any subject consent. The study will be conducted in accordance with Good Clinical Practice guidelines and all applicable country, state, and local regulations.

### Primary outcome measures

AIRFLOW-3 is the first interventional COPD trial with a primary objective of reduction in moderate or severe (hospitalized) exacerbations compared with optimal medical care alone. For the purpose of enrollment and follow-up in this study, a COPD exacerbation will be defined as a complex of respiratory events/symptoms (increase or new onset) of more than one of the following: cough, sputum, wheezing, dyspnea or chest tightness with at least one symptom with a duration of at least 3 days and requiring treatment with antibiotics and/or corticosteroids (moderate exacerbation) and including hospital admission or emergency room / acute care visit > 24 h in duration (severe exacerbation) [13]. COPD exacerbations will be determined by and treated at the discretion of the Investigator in accordance with guideline-based recommendations.

To assess the primary objective, the primary endpoint is a comparison of time-to-first event for moderate or severe COPD exacerbations between the active treatment arm and the sham control arm based on a log-rank test. Event timing will be based on the time from the date of randomization to the date of a patient’s first primary endpoint event, or to the close of the 12-month visit window for patients who do not experience a primary endpoint event. Patients who have not experienced a primary endpoint event and are lost to follow-up, or withdrawn, prior to the close of the 12-month visit window, will be censored at the date of their last known status.

### Secondary outcome measures

Secondary outcome measures will include comparisons between study arms of time to first respiratory-related hospitalization and time to first severe exacerbation at 12 months. Other prespecified secondary outcome measures include the difference between study and control group at 12 months for: quality of life (St. George’s Respiratory Questionnaire COPD version (SGRQ-C) scores, CAT scores, short form health survey (SF-36) scores), transitional dyspnea index (TDI), and changes in spirometric (FEV1 and FVC) and plethysmographic (RV) lung volume measures, see Table 1.

**Table 1:** Primary and Secondary Endpoints

	Time Point
Primary endpoint	
Time-to-first event:	Through 12 months
Moderate or Severe COPD Exacerbations	
Secondary endpoints	
Time-to-first event	Through 12 months
Severe COPD exacerbations	
Respiratory-related hospitalizations	
Changes in quality of life	Through 12 months
SGRQ-C	
CAT Responders	
SF-36 Change	
Changes in dyspnea	Through 12 months
Transition Dyspnea Index (TDI)	
Changes in lung function^a^	Through 12 months
FEV1	
FVC	
RV	

^a^Changes in lung function are measured using spirometry (FEV1 and FVC) and plethysmography (RV). Abbreviations: *CAT* COPD assessment test; *FEV1* forced expiratory volume in 1 s; *FVC* forced vital capacity; *IC* inspiratory capacity; *SF-36* short form health survey; *SGRQ-C* St. George’s Respiratory Questionnaire COPD version; *TLC* total lung capacity; *TDI* transition dyspnea indexes; *RV* residual volume

### Sub-studies

A sub-study on airway inflammatory biomarkers will be offered to randomized subjects at participating AIRFLOW-3 centers. Bronchial brushes will be collected at the time of the study procedure and during a second airway inspection at 6-months post-procedure. Three brushes from the right lower lobe segmental bronchi will be collected and analyzed. The study is exploratory in nature and gene expression changes after TLD will be based on transcriptome analysis; including differentially expressed genes, cluster analysis, and gene set enrichment [14].

### Patient recall and recruitment

Monthly phone-visit follow-ups are planned for months when an in-person follow-up visits do not occur. A memory aid to record daily symptoms of exacerbation and medications will be provided to support patient recall of changes in respiratory symptoms, medications, and any respiratory-related healthcare resource utilization for the first 12 months.

Study sites may advertise for local recruitment. Study information and/or slides for presentation can be provided to referring physicians upon request. IRB/EC approval of any materials to be used for direct patient recruitment will be obtained by the reviewing IRB/EC prior to use.

### Screening assessments

**Fig. 1:**
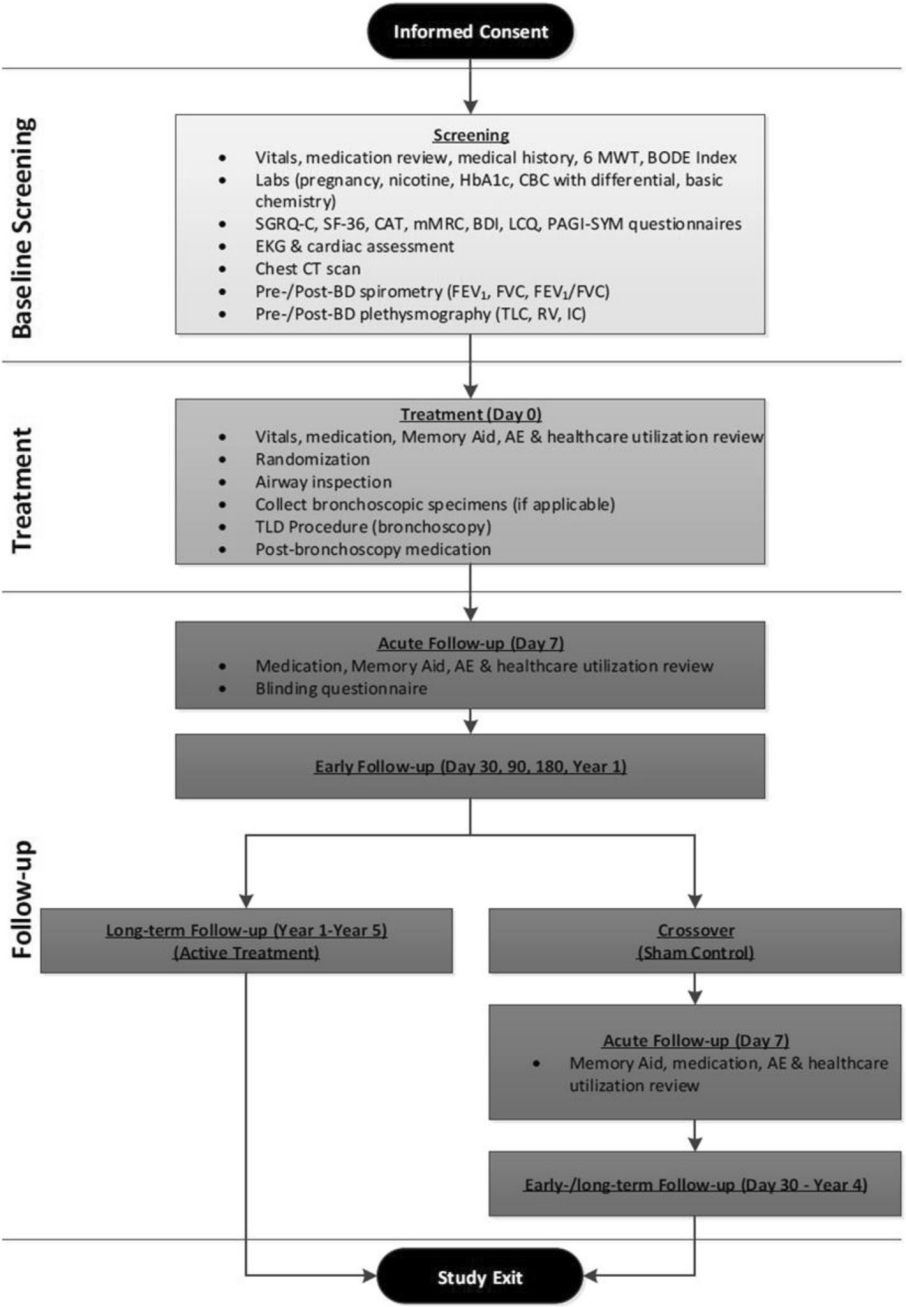
Participant flow through the study

**Fig. 2:**
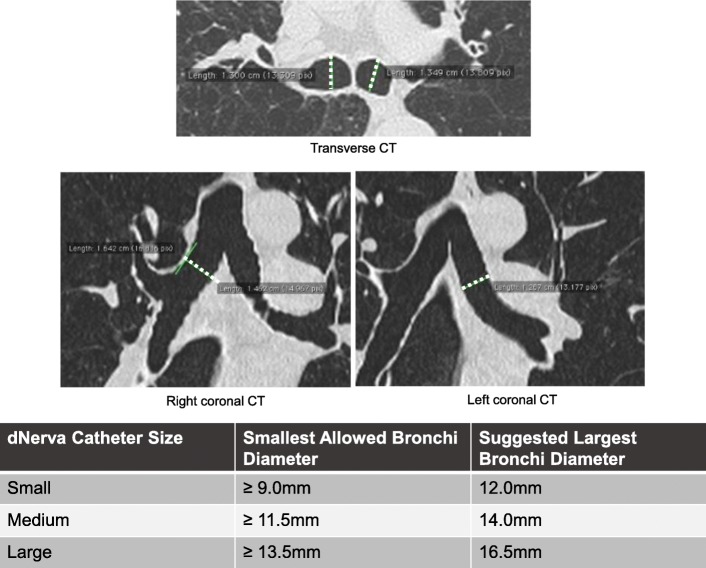
Example of transverse and coronal slice CT airway measurements. The patient’s computed tomography scan of the chest is reviewed prior to the procedure to re-confirm proper airway sizes and geometry. The right mainstem bronchial length must be ≥10 mm to ensure an adequate location for electrode placement. The diameters measured on the transverse scan and coronal scan (diameter indicated by white dotted lines on CT images) must be averaged to determine the appropriate catheter size for both mainstem bronchi

A summary of participant flow from consent to study exit is detailed in Fig. 1 and required testing and assessments are included in Additional file 1. Participants will be considered enrolled at the time of consent. Following consent, participants will undergo baseline screening to assess eligibility including medical history. Inclusion/exclusion criteria for the study are outlined in detail in Tables 2 and 3. Participants must have documented exacerbation history of at least 2 moderate COPD exacerbations or 1 severe COPD exacerbation in the 12-months prior to enrollment while on optimal maintenance COPD medications (minimum 12 months on LABA/LAMA therapy, or similar pharmacologic regimen). All participants will have an inspiratory chest CT scan submitted to the core lab for exclusionary review and confirmation of appropriate airway sizing prior to treatment (Fig. 2).

**Table 2:** Inclusion Criteria

1	Participants aged ≥40 and ≤ 75 years at the time of consent.
2	Women of childbearing potential must have a negative pregnancy test (blood or urine) pre-treatment and agree not to become pregnant for the duration of the study.
3	Smoking history of at least 10 pack years.
4	Non-smoking for a minimum of 2 months prior to consent and agrees to not smoke for the duration of the study. Negative nicotine test required, if participant is taking smoking cessation medication, patch, gum, etc., a quantitative test should be performed to assess if measured level of nicotine or cotinine is below study threshold(s).
5	Received a flu vaccination within the 12 months prior to consent and agrees to annual vaccinations for the duration of the study.
6	SpO2 ≥ 89% on room air at the time of screening.
7	CAT score ≥ 10 at the time of screening.
8	Diagnosis of COPD with 30% ≤ FEV1 < = 60% of predicted and FEV1/FVC < 70% (post-bronchodilator).
9	Documented history of ≥2 moderate COPD exacerbations or ≥ 1 severe COPD exacerbation leading to hospitalization in the 12 months prior to consent.
10	Documented history of taking at least LAMA and a LABA as regular respiratory maintenance medication for ≥12 months at the time of consent. Participants who have documented intolerance to LAMA and/or LABA but are taking a minimum of two regular respiratory maintenance medications (e.g., ICS/LABA) are eligible for participation. Participants who do not respond to LABA and LAMA maintenance inhaler therapy will be allowed to use nebulized bronchodilator therapy.
11	Recent participation in a formal pulmonary rehabilitation program should have occurred ≥3 months prior to consent; if participant is currently enrolled in a maintenance program, they agree to continue their current program through their 12-month follow-up visit.
12	Candidate for bronchoscopy in the opinion of the physician or per hospital guidelines. Examples of suitability of participant for bronchoscopy include, but are not limited to: cardiovascular fitness, ability of participant to be intubated, ability to oxygenate patient, absence of previously diagnosed high-grade tracheal obstruction, absence of uncorrectable coagulopathy (i.e. participant is unable to stop taking blood thinning medication, with the exception of aspirin, 7 days before and not restart until 7 days after the study procedure).
13	Willing, able, and agrees to complete all protocol required baseline and follow-up testing assessments including taking certain medications (e.g., azithromycin, prednisolone / prednisone).
14	Provided written informed consent using a form reviewed and approved by the IRB/EC.

Abbreviations: *CAT* COPD assessment test; *FEV1* forced expiratory volume in 1 s; *FVC* forced vital capacity; *ICS* inhaled corticosteroids; *IRB/EC* Institutional review board/ethics committee; *LABA* long-acting beta agonists; *LAMA* long-acting muscarinic antagonists

**Table 3:** Exclusion Criteria

1	BMI between < 18 or > 35.
2	Has an implantable electronic device.
3	Uncontrolled diabetes as evidenced by an HbA1c > 7%.
4	Pulmonary nodule thought to be at high risk of malignancy.
5	Malignancy treated with radiation or chemotherapy within 2 years of consent.
6	More than 3 respiratory-related hospitalizations within 1 year of consent.
7	Asthma as defined by the current GINA guidelines.
8	Patient has been previously diagnosed with a non-COPD lung disease or has a documented medical history of pneumothorax.
9	Clinically relevant bronchiectasis, defined as severe single lobe or multilobar bronchial wall thickening associated with airway dilation on CT scan leading to cough and tenacious sputum on most days.
10	Pre-existing diagnosis of pulmonary hypertension, defined as a sustained elevation of the systolic pulmonary artery pressure ≥ 25 mmHg at rest by right heart catheterization or estimated by echocardiogram to be > 40 mmHg.
11	Myocardial infarction within last 6 months, EKG with evidence of life-threatening arrhythmias or acute ischemia, pre-existing documented evidence of a LVEF < 45%, stage C or D (ACC/AHA) or Class III or IV (NYHA) congestive heart failure, or any other cardiac findings that make the participant an unacceptable candidate for a bronchoscopic procedure utilizing general anesthesia.
12	Known gastrointestinal motility disorder or previous abdominal surgical procedure on stomach, esophagus, or pancreas.
13	A GCSI total symptom score ≥ 18.0 (sum of PAGI-SYM questions 1–9) prior to treatment.
14	Any disease or condition that might interfere with completion of a procedure or this study (e.g., structural esophageal disorder, life expectancy < 3 years).
15	Prior lung or chest procedure. Segmentectomy for benign lesion or segmentectomy for non-recurrent cancer ≥2 years is allowed.
16	Daily use of > 10 mg of prednisone or its equivalent at the time of consent.
17	Recent (within 3 months of consent) opioid use.
18	Known contraindication or allergy to medications required for bronchoscopy or general anesthesia that cannot be medically controlled.
19	Screening chest CT scan reveals bronchi anatomy cannot be fully treated with available catheter sizes, presence of severe emphysema > 50%, lobar attenuation area or severe bullous disease (> 1/3 hemithorax) (as determined by the CT core lab using a single density mask threshold of − 950 HU) or site discovery of a mass that requires treatment.
20	In the opinion of the treating Investigator, use of the Nuvaira System is not technically feasible due to patient anatomy or other clinical finding.
21	Enrolled in another clinical trial that has not completed follow-up.

Abbreviations: *ACC/AHA* American College of Cardiology/American Heart Association; *BMI* body mass index; *GCSI* gastroparesis cardinal symptom index; *GINA* Global Initiative for Asthma; *LVEF* left ventricular ejection fraction; *NYHA* New York Heart Association; *PAGI-SYM* patient assessment of gastrointestinal disorders symptom severity index

A cardiac assessment, including an ECG and medical clearance for anesthesia, will be required as part of baseline screening. To exclude patients with gastrointestinal symptoms, the validated, patient-reported gastroparesis cardinal symptom index (GCSI) assessment [15] will be administered. Scores of ≥18.0 on this index will be exclusionary.

### Respiratory medication

Since the primary objective of this study compares TLD plus optimal medical care to optimal medical care alone, it will be important to document and control respiratory medications from the time of consent through the primary endpoint analysis period unless there is a drug specific adverse event requiring discontinuation. For the purposes of this study, optimal medical care is recommended per the GOLD 2019 guidelines [1]. Participants already taking an inhaled corticosteroid (ICS) or other classes of medication at the time of consent should continue taking them through the one-year study exit visit, to avoid potential confounding impact of medication changes. Randomization will be stratified to ensure an equal distribution of ICS patients in the sham and treatment arms. When necessary, changes in COPD medications are permitted for a legitimate medical need to protect the subject and will not be documented as a protocol deviation. All medication changes will be closely monitored and recorded for the duration of the study.

### Blinding and group allocations

The study blinding plan will be implemented at each of the sites to ensure that double-blinding is maintained throughout the 12-month follow-up period. Blinded (follow-up visits) and unblinded (study procedure) teams will be formed at each center. All sham and TLD procedures will be performed by a physician. Participants randomized to the sham group will undergo a sham TLD procedure with the Nuvaira lung denervation system (the esophageal balloon and dNerva® catheter will be placed and the balloon inflated but no fluoroscopy or radiofrequency (RF) energy will be delivered). Steps will be taken to ensure that the bronchoscopy suite is staged, and equipment manipulated in a manner that provides a similar total procedure experience regardless of treatment allocation. Of note, TLD yields no radiographically visible implants or treatment evidence. Following the 12-month follow-up period of double-blinding, participants in the sham group will be offered the opportunity to undergo TLD therapy, followed for up to 4 years and evaluated as a cross over group.

**Fig. 3:**
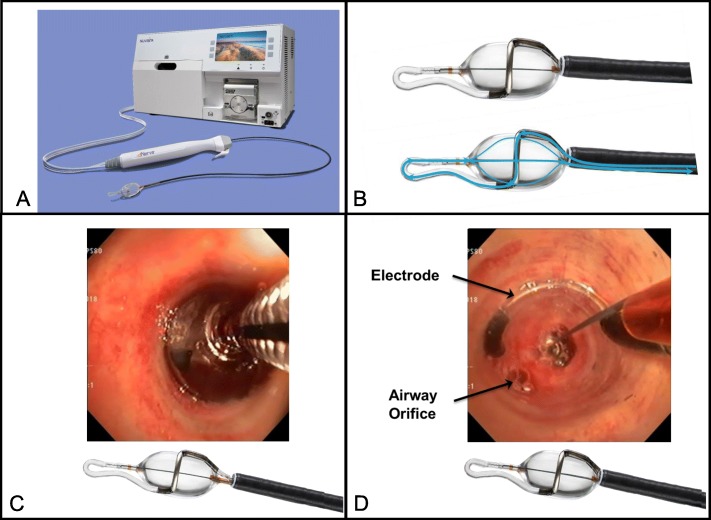
Image of the Nuvaira Console with the dNerva Catheter in bronchoscope and the expandable cooled balloon. **a** An image of the Nuvaira lung denervation system including the Nuvaira Console and dNerva Catheter. **b** The dNerva catheter is inserted through the working channel of a flexible bronchoscopes and inflated following positioning. Cooling fluid is circulated through the catheter by the console and provides the cooling that protects the airway wall during energy delivery (blue arrows indicate fluid flow). **c** During the procedure, the catheter is positioned in the mainstem bronchi and **d** visualization of the electrode positioning is confirmed by coupling the bronchoscope to the distal end of the catheter balloon

**Fig. 4:**
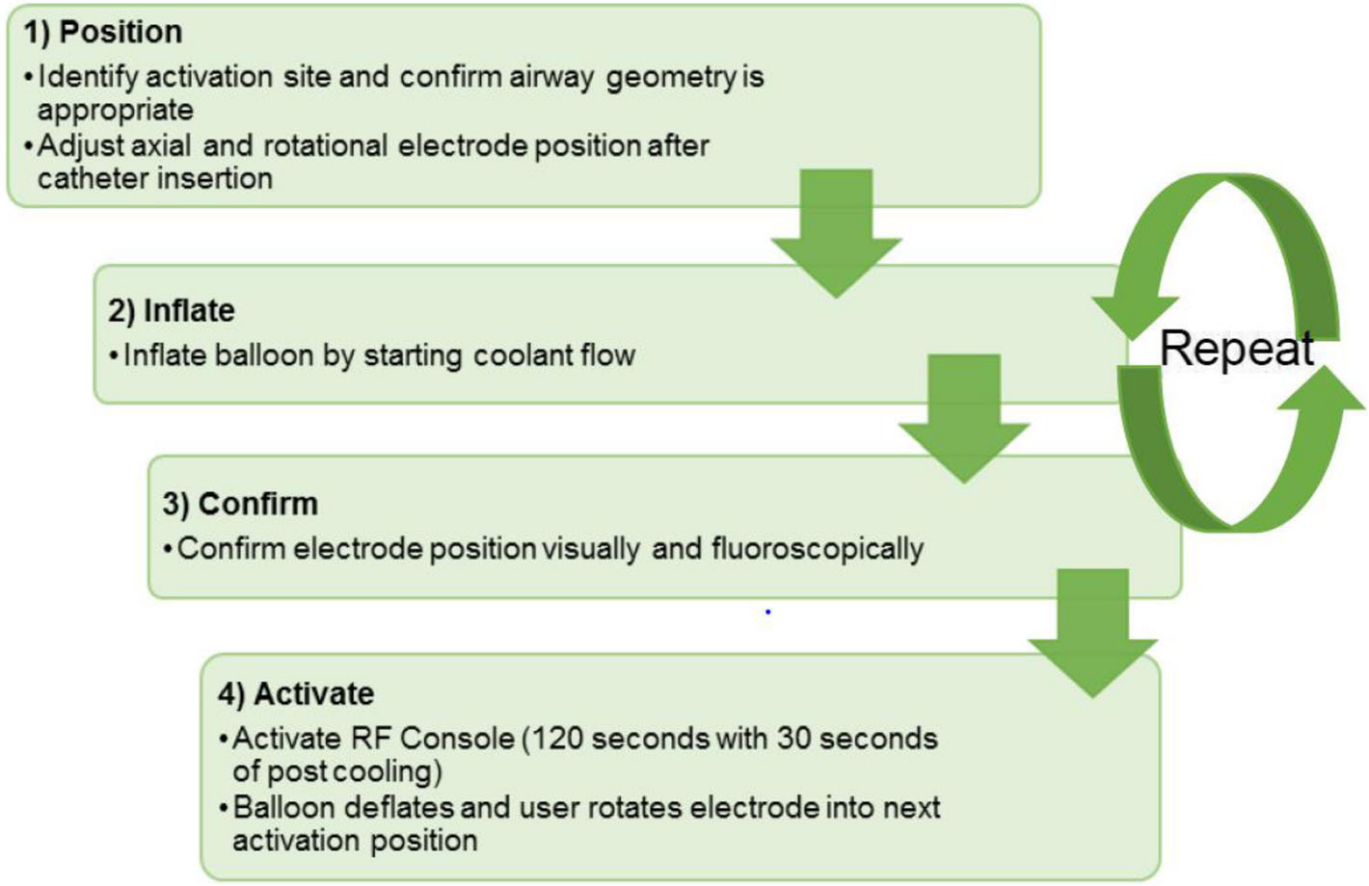
Key steps of the targeted lung denervation procedure. All four steps are repeated until the entire circumference of the first bronchus is treated to ablate the nerves. This is typically achieved in 4 activations. The catheter is then retracted, and the opposite main bronchus treated

The treatment group will undergo active TLD treatment with the Nuvaira lung denervation system (fluoroscopy and RF energy will be delivered). TLD is delivered via a dual-cooled balloon catheter as previously described, see Fig. 3 [10–12, 16]. The Nuvaira catheter is passed through the working channel of a 3.2 mm flexible bronchoscope and coupled with the bronchoscope. This provides direct visualization of the catheter, balloon, and electrode-tissue interface and guides proper axial positioning along the length of the bronchi. Fluoroscopy is used to guide the proper rotational positioning and the electrode distance from the esophagus. Complete circumferential treatment involves 4 quadrant rotations of the catheter in each main bronchus. Both lungs are treated in a single procedure, with an average of one or two catheters used, depending on airway dimension. The procedure steps are described in Fig. 4.

At each site, the first 3 enrolled participants will be treated with TLD therapy (no randomization) as “roll-in cases” designed to evaluate the procedural learning curve. These patients will undergo the same pre- and post-treatment assessments as randomized subjects but will be analyzed separately from the randomized cohort. All subjects will be followed for 5 years.

### Statistical analysis

Primary endpoint analyses will be conducted on the intent-to-treat (ITT), randomized population.

Secondary endpoints (including adverse events) will be based on a modified ITT population (all participants for whom the active procedure or the sham procedure is initiated, excluding roll-in procedure participants). One-sided statistical tests will be considered significant at *p*-values less than 0.025 while two-sided tests will be significant at p-values less than 0.05. The statistical test for the primary endpoint will be based on a log-rank test, comparing the survival distribution of the time-to-first event for the primary endpoint. The secondary endpoints will be tested using a combination of sequential gatekeeping procedure and Hommel adjustment to control for type I error rate.

Sample size for the study was driven by the number of primary endpoint events required to obtain adequate power and based on event-rate data from AIRFLOW-2. Assuming the percentage of participants with primary endpoint events through 12 months is 65 and 48.75% for the sham control and TLD groups respectively, a sample size of 400 will provide greater than 90% power based on a two-sided 0.05 alpha level log-rank test. An attrition rate of 10% at 12-months has been accounted for in the sample size calculation.

### Safety

Oversight of the overall conduct of the study is the responsibility of the Steering Committee, which includes, but is not limited to, dissemination of data (including publications), recommendations of the independent data monitoring committee (DMC), and ongoing review of safety data. The DMC will provide independent monitoring of the study. The DMC will be charged with monitoring the overall study for safety, invoking study stopping rules, and for auditing the quality of the data. Treatment safety will be assessed by monitoring the incidence of all adverse events (AEs), serious adverse events (SAEs), and all unanticipated adverse device effects (UADEs) from randomization through 12 months. An independent Clinical Events Committee will adjudicate all AEs [1].

Long-term safety will be assessed by monitoring the incidence of a prospectively defined subset of important respiratory, cardiovascular, and gastrointestinal SAEs, and all-cause mortality out to 5 years. Sham control participants who are treated after the first year of follow-up will be included in the assessment of long-term safety.

### Study timeline

The study will begin in May of 2019 and final follow-up of the primary endpoint is expected to be completed in August of 2022.

### Study organization

This study was designed and guided by the study steering committee which consisted of 2 principal investigators and 5 physicians, working in academic hospital settings. Electronic data are collected at the study sites; data transfer, management and storage, quality control rest on Nuvaira, Inc. The AIRFLOW-3 trial is fully sponsored by Nuvaira, Inc. USA.

## Discussion

Continued exacerbation events in guideline treated patients remains a therapeutic challenge in COPD management [17–19]. Acute COPD exacerbations are associated with a rapid decline in lung function and with impaired survival [20], mortality in the year following a severe hospitalized exacerbation is estimated to be as high as 21% [21]. In the US, exacerbation-related costs represent the highest proportion of total COPD costs, across all levels of disease severity [22].

This study requires that patients be taking a minimum of two bronchodilators (i.e. stable GOLD guideline drug therapy) for 12 months prior to randomization and strongly recommends no maintenance therapy changes during the 12-month period following randomization. Maintenance add-on therapies (i.e., ICS, PDE4 inhibitors and azithromycin) are allowed at the discretion of the treating physician. All patient drug use will be tracked and recorded throughout the randomization period. Additionally, patients using an ICS will be stratified evenly between the treatment and sham groups [23].

Pulmonary rehabilitation (PR) improves dyspnea, health status, and exercise tolerance in stable patients, although PR appears to have no measurable impact on the risk of COPD exacerbation [24]. However, PR is under-utilized in COPD, particularly in the US [25]. Therefore, a requirement for PR program completion, while ideal, would result in a potential impediment to enrollment for significant numbers of otherwise qualified subjects. AIRFLOW-3 will thus record patient experience with PR (naïve or past participation) at baseline and throughout the trial, but will not require pulmonary rehabilitation as an inclusion criterion. Rather, subjects will be stratified based on their prior PR experience.

Time-to-first event analysis is considered the most robust way to measure COPD exacerbations in clinical studies because it is unlikely to be affected by early patient exits and associated missing data [26]. Analysis of the proportion of patients experiencing at least one exacerbation event is also important particularly in the context of individual risk-reward preferences. Recently published, large-scale randomized controlled trials targeting reduction in COPD exacerbation have documented that up to 65% of patients experience at least one moderate or severe exacerbation over 12 months of study follow-up [19, 23]. Percent reduction in total number of exacerbations can generate a large between-group difference skewed by a small number of patients with a disproportionately high number of events [27]. Therefore, the primary endpoint for AIRFLOW-3 is the time-to-first event analysis of the proportion of patients experiencing one or more moderate or severe exacerbations, comparing the active treatment (TLD) to the sham-control arm.

Increasingly, the COPD literature examining the effects of pharmacological treatment suggests that there are only modest alterations in secondary outcomes associated with clinically meaningful reductions in exacerbations [23, 28]. The SPARK trial comparing dual with single maintenance bronchodilator therapy (tiotropium) reported a 12% reduction in all moderate or severe exacerbation events favoring dual therapy, yet clinically unimportant changes in FEV_1_ (increased 60 vs. 80 mL) and SGRQ-C (decreased − 1.7 vs. -3.1) between groups [28]. In IMPACT, the 6.8% relative reduction in the proportion of patients with a moderate or severe COPD exacerbation was associated with differences of 54 mL in FEV_1_ and a − 1.8 in SGRQ-C between groups [23]. Such discrepancies between changes in exacerbation events and changes in baseline symptoms or lung function is biologically and medically consistent with a potential mechanism of TLD, that being disruption of reflex airway reactivity. AIRFLOW-3 will explore the impact of treatment on secondary outcomes such as FEV_1_ and SGRQ-C.

In conclusion, the AIRFLOW-3 trial will evaluate the efficacy of TLD to reduce moderate or severe COPD exacerbations beyond optimal medical treatment. Earlier-phase trials have demonstrated feasibility and a positive safety profile of TLD in COPD patients out to 3-years post-treatment. The target population is GOLD Group D patients, who suffer persistent symptoms and exacerbations despite optimal guideline-directed therapy, defining an unmet medical need requiring a novel therapeutic solution.

## Supplementary information

**Additional file 1.** Required Testing and Assessments.

## Data Availability

Not applicable.

## Abbreviations

AE: Adverse event
CAT: COPD assessment test
COPD: Chronic obstructive pulmonary disease
DMC: Data monitoring committee
EC: Ethics committee
GCSI: Gastroparesis cardinal symptom index
GOLD: Global Initiative for Chronic Obstructive Lung Disease
ICS: Inhaled corticosteroid
IRB: Institutional review board
ITT: Intent-to-treat
LABA: Long-acting ß-agonist
LAMA: Long-acting muscarinic antagonist
PFT: Pulmonary function testing
PR: Pulmonary rehabilitation
TLD: Targeted lung denervation
