# Supplementary material for: Design for a multicenter, randomized, sham-controlled study to evaluate safety and efficacy after treatment with the Nuvaira® lung denervation system in subjects with chronic obstructive pulmonary disease (AIRFLOW-3)
Source: BMC Pulm Med. 2020 Feb 13;20:41. doi: 10.1186/s12890-020-1058-5 (PMC7020591; doi:10.1186/s12890-020-1058-5)
Supplement: Supplementary file 1 — Additional file 1. Required Testing and Assessments. [file 12890_2020_1058_MOESM1_ESM.docx]

Supplemental Appendix 1. Required Testing and Assessments.

|  | | | | | | | | | | |
| --- | --- | --- | --- | --- | --- | --- | --- | --- | --- | --- |
|  | **Baseline Screening** | **Treatment** | **Early Follow-Up**  *(6-week lock-out period required between resolution of a respiratory event and any study visit which requires PFT)* | | | | | | | |
| Visit | Screening | Day 0 | Day 7 | Month 1 | Month 2 | Month 3 | Month 4 & 5 | Month 6 | Month 7 & 8 | Month 9 |
| Visit Type | Office | Hospital | Phone | Office | Phone | Office | Phone | Office | Phone | Office |
| Visit Window | - | ≤30 days  After Screening | +/- 2 days | +/- 10 days | +/- 10 days | +/- 10 days | +/- 10 days | +/- 10 days | +/- 10 days | +/- 10 days |
| **Visit Number** | **1** | **2** | **3** | **4** | **5** | **6** | **7-8** | **9** | **10-11** | **12** |
| Informed Consent | **✓** |  |  |  |  |  |  |  |  |  |
| SF-36 | **✓** |  |  |  |  |  |  | **✓** |  |  |
| BDI/TDI**^*^** | **✓** |  |  |  |  |  |  | **✓** |  |  |
| LCQ | **✓** |  |  |  |  |  |  | **✓** |  | **✓** |
| SGRQ-C | **✓** |  |  | **✓** |  | **✓** |  | **✓** |  | **✓** |
| CAT | **✓** |  |  | **✓** |  | **✓** |  | **✓** |  | **✓** |
| mMRC | **✓** |  |  | **✓** |  | **✓** |  | **✓** |  | **✓** |
| PAGI-SYM | **✓** |  |  | **✓** |  | **✓** |  | **✓** |  | **✓** |
| Blinding Questionnaire |  |  | **✓** |  |  |  |  |  |  |  |
| Memory Aid Review |  | **✓** | **✓** | **✓** | **✓** | **✓** | **✓** | **✓** | **✓** | **✓** |
| Medication Review | **✓** | **✓** | **✓** | **✓** | **✓** | **✓** | **✓** | **✓** | **✓** | **✓** |
| Resource Utilization Review |  | **✓** | **✓** | **✓** | **✓** | **✓** | **✓** | **✓** | **✓** | **✓** |
| AE Review |  | **✓** | **✓** | **✓** | **✓** | **✓** | **✓** | **✓** | **✓** | **✓** |
| Physical Exam | **✓** | **✓** |  | **✓** |  | **✓** |  | **✓** |  | **✓** |
| 6 MWT | **✓** |  |  |  |  |  |  |  |  |  |
| BODE Index | **✓** |  |  |  |  |  |  |  |  |  |
| EKG & Cardiac Assessment | **✓** |  |  |  |  |  |  |  |  |  |
| Labs**^**^** | **✓** |  |  |  |  |  |  |  |  |  |
| Pre-/Post-BD Spirometry^†^ | **✓** |  |  |  |  | **✓** |  | **✓** |  | **✓** |
| Pre-/Post-BD Plethysmography^††^ | **✓** |  |  |  |  |  |  | **✓** |  |  |
| Chest CT Scan**^***^** | **✓** |  |  |  |  |  |  |  |  |  |
| Airway Inspection |  | **✓**^§^ |  |  |  |  |  | **✓**^§§^ |  |  |
| TLD Procedure |  | **✓** |  |  |  |  |  |  |  |  |
| **Inflammatory Markers**^†††^ |  | **✓** |  |  |  |  |  | **✓** |  |  |

| **Required Testing & Assessments** **(continued)** | | | | | | | | | | |
| --- | --- | --- | --- | --- | --- | --- | --- | --- | --- | --- |
|  | **Long-term Follow-Up**  *(6-week lock-out period required between resolution of a respiratory event and any study visit which requires PFT)* | | | | | | | | | |
| Visit | Month 10 & 11 | Month 12 | Month 15, 18 & 21 | Year 2 | Year 2.5 | Year 3 | Year 3.5 | Year 4 | Year 4.5 | Year 5 |
| Visit Type | Phone | Office | Phone | Office | Phone | Office | Phone | Office | Phone | Office |
| Visit Window | +/- 10 days | +/- 10 days | +/-35 days | +/-35 days | +/-35 days | +/-35 days | +/-35 days | +/-35 days | +/-35 days | +/-35 days |
| **Visit Number** | **13-14** | **15*^§§§^*** | **16-18** | **19** | **20** | **21** | **22** | **23** | **24** | **25** |
| SF-36 |  | **✓** |  | **✓** |  | **✓** |  | **✓** |  | **✓** |
| TDI**^*^** |  | **✓** |  |  |  |  |  |  |  |  |
| LCQ |  | **✓** |  | **✓** |  | **✓** |  | **✓** |  | **✓** |
| SGRQ-C |  | **✓** |  | **✓** |  | **✓** |  | **✓** |  | **✓** |
| CAT |  | **✓** |  | **✓** |  | **✓** |  | **✓** |  | **✓** |
| mMRC |  | **✓** |  | **✓** |  | **✓** |  | **✓** |  | **✓** |
| PAGI-SYM |  | **✓** |  | **✓** |  | **✓** |  | **✓** |  | **✓** |
| Blinding Questionnaire |  | **✓** |  |  |  |  |  |  |  |  |
| Memory Aid Review | **✓** | **✓** | **✓** | **✓** |  |  |  |  |  |  |
| Medication Review | **✓** | **✓** | **✓** | **✓** | **✓** | **✓** | **✓** | **✓** | **✓** | **✓** |
| Resource Utilization Review | **✓** | **✓** |  |  |  |  |  |  |  |  |
| AE Review | **✓** | **✓** | **✓** | **✓** | **✓** | **✓** | **✓** | **✓** | **✓** | **✓** |
| Physical Exam |  | **✓** |  | **✓** |  | **✓** |  | **✓** |  | **✓** |
| 6 MWT |  | **✓** |  |  |  |  |  |  |  |  |
| BODE Index |  | **✓** |  |  |  |  |  |  |  |  |
| Labs**^**^** |  | **✓** |  | **✓** |  | **✓** |  | **✓** |  | **✓** |
| Pre-/Post-BD Spirometry^†^ |  | **✓** |  | **✓** |  | **✓** |  | **✓** |  | **✓** |
| Pre-/Post-BD Plethysmography^††^ |  | **✓** |  | **✓** |  | **✓** |  | **✓** |  | **✓** |
| *^*^ BDI at baseline “Screening Visit” and TDI at all other designated time points.*  *^**^ Screening (all subjects) & 12-Month Follow-up (all subjects, except “roll-in” subjects): HbA1c (diabetics only), nicotine test (blood, urine or saliva), pregnancy test (women of child-bearing potential only), CBC with differential, basic chemistry. 12-Month Follow-up (“roll-in” subjects): nicotine test (blood, urine or saliva). Year 2, 3, 4, & 5 (all subjects): nicotine test (blood, urine or saliva).*  *^***^CT after reversibility testing (administration of bronchodilator) when both tests are performed on the same day.*  ^†^ *FEV_1_, FVC, FEV1/FVC required. Initial testing at 10:00 preferred; follow-up testing within ± 2-hour window of baseline test time. Study questionnaires should be completed prior.*  ^††^ *TLC, RV, IC required. Initial testing at 10:00 preferred; follow-up testing within ± 2-hour window of baseline test time. Study questionnaires should be completed prior.*  ^†††^  *Sub-study requirement at participating sites (highlighted in blue above). Roll-in and crossover subjects are not eligible to participate in the inflammatory biomarker sub-study.*  ^§^ *Required twice, both at the beginning and at the end of the index study procedure for all subjects (roll-in and randomized; both Active Treatment and Sham Control procedures).*  ^§§^ *Not required to be performed under general anesthesia.*  ^§§§^ *Unblinding to occur at the close of 12-month follow-up visit window. Unblinding questionnaire should be administered just prior to unblinding.* | | | | | | | | | | |
